# Pioneer *Arabidopsis thaliana* spans the succession gradient revealing a diverse root-associated microbiome

**DOI:** 10.1186/s40793-023-00511-y

**Published:** 2023-07-19

**Authors:** Vera Hesen, Yvet Boele, Tanja Bakx-Schotman, Femke van Beersum, Ciska Raaijmakers, Ben Scheres, Viola Willemsen, Wim H. van der Putten

**Affiliations:** 1 Cluster of Plant Developmental Biology, Laboratory of Molecular Biology, Wageningen University, Droevendaalsesteeg 1, Wageningen, 6708 PB the Netherlands; 2 Department of Terrestrial Ecology, Netherlands Institute of Ecology (NIOO-KNAW), Droevendaalsesteeg 10, Wageningen, 6700 AB the Netherlands; 3 Plant Ecology and Nature Conservation Group, Wageningen University, Droevendaalsesteeg 3a, Wageningen, 6708 PB the Netherlands; 4 Department of Biotechnology, Rijk Zwaan Breeding B.V., Eerste Kruisweg 9, Fijnaart, 4793 RS the Netherlands; 5 Laboratory of Nematology, Wageningen University, Droevendaalsesteeg 1, Wageningen, 6708 PB the Netherlands

**Keywords:** Secondary succession, Soil, Bacteria, Fungi, Chronosequence

## Abstract

**Background:**

Soil microbiomes are increasingly acknowledged to affect plant functioning. Research in molecular model species *Arabidopsis thaliana* has given detailed insights of such plant-microbiome interactions. However, the circumstances under which natural *A. thaliana* plants have been studied so far might represent only a subset of *A. thaliana’s* full ecological context and potential biotic diversity of its root-associated microbiome.

**Results:**

We collected *A. thaliana* root-associated soils from a secondary succession gradient covering 40 years of land abandonment. All field sites were situated on the same parent soil material and in the same climatic region. By sequencing the bacterial and fungal communities and soil abiotic analysis we discovered differences in both the biotic and abiotic composition of the root-associated soil of *A. thaliana* and these differences are in accordance with the successional class of the field sites. As the studied sites all have been under (former) agricultural use, and a climatic cline is absent, we were able to reveal a more complete variety of ecological contexts *A. thaliana* can appear and sustain in.

**Conclusions:**

Our findings lead to the conclusion that although *A. thaliana* is considered a pioneer plant species and previously almost exclusively studied in early succession and disturbed sites, plants can successfully establish in soils which have experienced years of ecological development. Thereby, *A. thaliana* can be exposed to a much wider variation in soil ecological context than is currently presumed. This knowledge opens up new opportunities to enhance our understanding of causal plant-microbiome interactions as *A. thaliana* cannot only grow in contrasting soil biotic and abiotic conditions along a latitudinal gradient, but also when those conditions vary along a secondary succession gradient. Future research could give insights in important plant factors to grow in more ecologically complex later-secondary succession soils, which is an impending direction of our current agricultural systems.

**Supplementary Information:**

The online version contains supplementary material available at 10.1186/s40793-023-00511-y.

## Background

Soil microbiomes play an important role in plant performance through feedback interactions between plant, biotic and abiotic soil properties. Soil microbes are able to affect a wide variety of plant processes such as belowground and aboveground defence responses, nutrient uptake, and flowering, thereby affecting plant growth and fitness [1–4]. In the past decades, plant-microbiome studies identified plant-microbiome interactions and plant core microbiomes in an array of wild plants and crop species including the molecular model plant, *Arabidopsis thaliana*. The use of this plant species enabled to capitalise extensive knowledge on plant physiology, genetics and molecular biology exemplified by numerous studies that provided detailed insights on plant-microbiome interactions [5–8]. Yet, there are still many aspects to consider, such as the circumstances under which *A. thaliana* plants and their root-associated microbiome have been studied so far.

Mechanistic studies on the selection strategies and composition of *A. thaliana* root-associated microbiomes often used well-known (lab) accessions or mutants. This approach in combination with the use of soil inocula from mostly ruderal and agricultural sites demonstrated that *A. thaliana* has some selection capacity for its rhizosphere and core microbiome [9–12]. However, the composition of *A. thaliana’s* root-associated and especially rhizosphere microbiome strongly depends on taxa that are present in the microbiome of the local bulk soil [10, 13]. This dependency underlines the importance of understanding the composition of the natural *A. thaliana* microbiome as the current studies might not reflect the full belowground biological diversity to which this species can be exposed.

Various studies examined soil communities of natural populations of *A. thaliana* in their local environment [14–17]. In most of these studies they focused on disturbed ecosystems, such as road verges, agricultural fields, or otherwise managed sites. It has been shown that disturbances and human management affected characteristics of the soil microbiome such as bacterial and fungal community composition, abundance, and functioning [18–21]. These disturbed ecosystems are in accordance with the ecology of *A. thaliana*, known as an opportunistic weed and pioneer colonizer of poor, ruderal soils. This is in line with the classification of *A. thaliana* as an early succession plant species, having a rapid life cycle and high levels of phenotypic plasticity [22, 23]. This early succession strategy is in contrast with those of more competitive mid and late succession plant species that sequentially replace early succession plant species after several years without disturbance [24].

Interestingly, in a recent study they transplanted *A. thaliana* in a 20 years-old abandoned agricultural field that was in a relatively late succession stage of soil community development [25]. This demonstrates *A. thaliana’s* ability to grow in a soil with a more complex ecology than was previously considered. Inspecting additional (former) agricultural fields in the same region showed that *A. thaliana* populations were common on early, mid, and late succession sites following soil disturbance by local animal activity (wild boars, hares, ants). This succession gradient spanning almost 40 years of land abandonment and soil development, suggests that, although considered a pioneer species, *A. thaliana* can appear and grow along a succession gradient with an inherently increasing soil microbiome complexity [26, 27]. It is yet unknown what this means for the microbiome composition on *A. thaliana*’s roots.

In addition to soil ecological context, climate is another factor of importance in plant-microbiome studies. In a recent study they looked into the soil microbiome of natural *A. thaliana* populations in various natural sites in Europe and revealed that in the *A. thaliana* rhizosphere the bacterial community composition was mostly dependent on the local soil. On the contrary, fungal community composition and plant performance were mostly affected by climatic conditions [28]. The effect of climate on plant performance is in line with previous studies where they showed how climate strongly affects natural selection and adaptation in *A. thaliana* [29–31]. As *A. thaliana* is native to Western Eurasia, it occurs naturally in a wide range of climatic conditions [32]. Studies in context of *A. thaliana* and its local soil microbiome on smaller geographic scales are rare to our knowledge and it remains an open question how much the local soil microbiome of *A. thaliana* can vary when studying this species in the absence of major climatic differences.

The aim of the present study was to elucidate the composition of the natural root-associated microbiome of *A. thaliana* in a secondary succession gradient from agricultural fields to long-term abandoned fields. For this, we used a well-studied Veluwe chronosequence located at the centre of the Netherlands which has all sites on the same sandy parental soil material [26, 27, 33, 34]. The chronosequence consists of a collection of sites that are either road verges, agricultural fields or fields that were taken out of agricultural production during the past 36 years (considered mid to late succession). We sampled a total of 11 sites comprised of different succession classes within 20 km^2^ and sequenced the bacterial and fungal communities and measured the soil abiotic composition. We discovered that both the abiotic soil conditions and the root-associated soil of *A. thaliana* can be highly diverse at a more local scale, and this variation is in accordance with the succession class of the site. Therefore, when studied in the same climatic conditions, *A. thaliana* plants can be exposed to a much wider variation of soil microbiome conditions along a successional gradient than is currently presumed, from arable fields to succession classes representing more than 40 years of land abandonment.

## Methods

### Experimental set-up and sampling

In May and June 2018 at the end of their flowering season, 51 dry seed-carrying *Arabidopsis thaliana* plants were collected from 11 different sites in the Veluwe region in the Netherlands (Additional file 1). The 11 sites represented 4 succession classes: 2 agricultural fields, 3 former agricultural mid succession fields, 4 former agricultural late succession fields and 2 road verges. The former agricultural fields were taken out of production 13 to 36 years prior to sampling. The agricultural sites, mid succession sites and late succession sites form a gradient while road verge sites acted as an outgroup representing a semi natural system. Per site 2–6 plants were sampled. More details on the sites and sampling methodology are presented in Additional file 2 which includes a map of the sampling locations (Fig. S1) and illustrative pictures of the sites (Fig. S2). For soil sampling, the plant root system was excavated with the surrounding bulk soil and placed on a flat surface. First the root-associated soil, which after shaking remained tightly adhered to the root, was collected. Using surface sterilized tools, 2 gram of root-associated soil was scraped of the roots and stored in sterile Eppendorf tubes to prevent contamination. Second, 200 gram of bulk soil surrounding the root system was collected in plastic bags. After sampling, the root-associated soil was stored at -80 °C prior to DNA extraction and bulk soil was stored at 4 °C prior to chemical analysis.

### Soil chemical analysis

The bulk soil was dried at 40 °C and sieved using a 1.4 mm mesh to remove plant material and other debris prior to chemical analysis. Soil Organic Matter (SOM) content (g/100 g dry soil) was measured using Loss On Ignition (LOI) method. Samples were dried for 16 h at 105 °C and then placed in a muffle furnace for 7 h at 430 °C. Soil organic matter content was calculated as the weight difference between samples heated at 105 and 430 °C, respectively. PO_4_-P content (mg/kg dry soil) was determined using the P-Olsen method (0.5 M NaHCO_3_ extraction, pH 8.5, 1:20 w/v) [35] for determination of plant-available orthophosphate and was measured using the AutoAnalyser SEAL QuAAtro Segmented Flow Analysis (SFA) system [36] (Beun de Ronde, Abcoude). Prior to CN analysis the dried and sieved soil was ground to a fine dust. Between 5000 and 6000 µg of ground soil was weighed and wrapped in a small tin-foil capsule. Carbon (C) and Nitrogen (N) content as a percentage of dry soil was measured using the micro-Dumas method with the Thermo Scientific FlashEA 1112 ElementAnalyser (Interscience, Breda). C:N ratio was calculated as percentage C divided by percentage N. Raw data is provided in Additional file 3.

### DNA extraction and amplification

The root-associated soil was used for sequencing the bacterial and fungal community composition. In the soil samples, there was residual plant material present and this was not removed prior to DNA extraction. DNA was extracted using approximately 0.25 gram of soil and the DNeasy PowerSoil Pro Kit (Qiagen) according to the manufacturer’s instructions. Bacterial community composition was determined by amplifying the 16 S rRNA gene (V4 region) using 515 F and 806R primers [37]. The fungal community composition was determined by amplifying the internal transcribed spacer 2 (ITS2) region using the ITS9 (forward) [38] and ITS4 (reverse) primers [39]. Both the 806R and ITS4 reverse primers included a twelve base barcode sequence to support multiplexing of samples (Additional file 4). For amplification, polymerase chain reactions (PCRs) were carried out with reaction mixtures containing 15.6 µl MQ, 2.5 µl FastStart high fidelity reaction buffer with 18 mM MgCl2 (10× concentrated), 2.5 µl dNTP’s (2µM), 0.15 µl Fast start high fidelity PCR system (Roche), 1 µl MgCl2 (25µM), 1.25 µl BSA (4 mg/ml) and 0.5 µl of forward primer (10µM), 0.5 µl of tagged reverse primer (10µM) and 1 µl genomic DNA. The PCR conditions were as follows: an initial denaturation step of 94 °C for 5 min, 35 cycles of 45 s at 94 °C, 60 s at either 50 °C (bacterial sequences) or 54 °C (fungal sequences) and 90 s at 72 °C, followed by a final extension step for 10 min at 72 °C. PCR products were purified using Agencourt AMPurebeads (Beckman Coulter) with 1:0.7 ratio of product to beads. The concentration of purified products was checked with fragment analysis (Standard Sensitivity NGS Fragment Analysis kit: 1-6000 bp, Advanced Analytical Technologies). Finally, 30 ng of amplicons per sample were processed by BGI (Shenzhen, China) by 250 bp paired-end sequencing with Illumina MiSeq.

### Sequence data processing and preparation

After sequencing, the raw sequencing data returned from BGI as filtered 250 bp reads in fastq format. BGI filtered the adaptor sequences, low-quality reads, and contaminations from raw reads. Besides the bacterial and fungal sequences, raw reads also contained reads from other kingdoms. Resulted sequences were submitted to European Nucleotide Archive under accession PRJEB59993. For unknown reasons two samples (MV-5 16 S rRNA gene data and AR1-3 ITS2 region data) had an extremely low number of reads. Both samples were eliminated by filtering out all samples with less than 1000 reads. The raw 16 S rRNA gene reads were processed with the DADA2 pipeline (v1.18.0) with default settings [40]. Processing included merging of the sequences, filtering, and removal of chimeras. Taxonomy was assigned by comparing the reads to the Silva 16 S rRNA gene database (v138.1) using the species extension Silva_Species [41]. DADA2 output was an amplicon sequence variant (ASV) abundance table. The raw ITS2 region reads were processed with the PIPITS pipeline (v2.7) with default settings [42]. PIPITS merged the paired-end reads, performed quality filtering, and assigned taxonomy of the sequences by comparing them to the UNITE fungal ITS2 region database (v8.2 [43]) with a 97% sequence similarity threshold [44]. Additionally, operational taxonomic units (OTUs) from other kingdoms than fungi were removed by PIPITS. PIPITS output was an OTU abundance table. Both 16 S rRNA gene and ITS2 region abundance table output files were transformed into csv files with ASV/OTU code respectively, species taxonomy (kingdom, phylum, class, order, family, genus and species) and sequence abundance counts. For 16 S rRNA gene data, ASVs from other kingdoms than bacteria were removed from the data afterwards.

### Data analysis

Three main data sets were generated: abiotic soil factor data (SOM, PO_4_-P, C%, N% and C:N ratio) with 51 samples, bacterial abundance counts with 50 samples (missing one late succession class sample) and fungal abundance counts with 50 samples (missing one agricultural succession class sample). The samples have a nested structure as there are a number of replicates per site, and each site is assigned to a succession class (Additional file 1). To account for the structure in the data, we nested site in succession class in the statistical models of analysis of variance (ANOVA), multivariate analysis of variance (MANOVA), and permutated multivariate analysis of variance (PERMANOVA). In addition, we used type-II (hierarchical) sum of squares in the ANOVA to account for the unbalanced data. All analyses were conducted using R (R Core Team, 2022) and implemented in RStudio (v.2022.12.0.353). Significance is indicated as follows: * *P* < 0.05; ** *P* < 0.01; *** *P* < 0.001.

### Abiotic soil analysis

The effect of succession class on abiotic soil factors was estimated using type-II ANOVA combined with Tukey’s Honest Significant Difference (Tukey HSD) as *post hoc* test. Principal component analysis (PCA) was used to analyse the combined abiotic soil factors and identify succession class clusters. The effect of succession class on the combined abiotic soil factors was estimated using a MANOVA. To follow up, a linear discriminant analysis (LDA) was used to see which succession classes differ most from one another. The LDA maximized the variance of the combined abiotic soil factors between succession classes while minimizing the within succession class variance of the combined abiotic soil factors. C:N ratio was excluded from the multivariate analysis (PCA, MANOVA and LDA) as it was not measured independently.

### Bacterial and fungal analysis

Before further analysis we determined the rarefaction curves using bacterial and fungal abundance count data to determine the relationship between the number of reads and number of observed ASVs/OTUs [45]. Prior to assessing the species richness (alpha diversity) we rarefied the bacterial data to 30,000 reads and fungal data to 10,000 reads. Bacterial samples MO_3 (late succession) and TW2_6 (road verge) and fungal samples AR1_6 (agricultural), DK_1 (late succession), and MO_1 (late succession) were omitted from species richness analysis due to insufficient sequence depth. Using the rarefied data, Shannon *H* index was calculated to assess the species richness and the resulting values were subjected to type-II ANOVA combined with Tukey HSD as *post hoc* test to compare succession class effects [46]. For the remainder of analysis, we normalized the bacterial and fungal abundance count data by cumulative sum scaling (CSS) to correct for sampling depth and library size of the samples but keeping a variation in total counts between samples [47]. We assessed the number of unique and overlapping bacterial ASVs and fungal OTUs between the succession classes using Venn diagrams. Percentile phyla abundances were calculated using the CSS normalized abundance counts per sample. Based on the highest CSS normalized read number we determined the top 10 most abundant bacterial ASVs and fungal OTUs and calculated their relative abundance per succession class (read number divided by total read number in succession class) (Additional file 6). Between-sample (beta) diversity of bacterial and fungal communities was determined by calculating Bray-Curtis dissimilarity matrices. Non-metric multidimensional scaling (NMDS) was used to visualize the effect of succession class on the bacterial and fungal community composition [48, 49]. Dimensions were initialized at 2, but increased when stress levels exceeded 0.2. The effect of succession class on bacterial and fungal community composition was estimated using a PERMANOVA with 999 permutations [50, 51]. Pairwise comparisons between succession classes were carried out using pairwise PERMANOVA with 999 permutations and Benjamini-Hochberg correction for multiple testing.

### Integrated abiotic, bacterial and fungal analysis

The independent abiotic soil factors (SOM, PO_4_-P, C% and N%) were scaled to standardize the data and allow direct comparisons between the soil factors. This was done by calculating the mean and standard deviation of each abiotic soil factor and subsequently each measurement was subjected to removing the respective mean and dividing by the respective standard deviation. The scaled data was then used to calculate a Euclidian distance matrix. We used this abiotic matrix (based on Euclidian distance) and previously calculated fungal and bacterial matrices (based on Bray-Curtis distances) for further analysis. The correlation between the abiotic matrix and either the bacterial or the fungal matrix was tested using a Mantel test with Pearson correlation and 999 permutations [52]. A Canonical Correspondence Analysis (CCA) was used to relate either the bacterial or the fungal matrix to the abiotic soil factors [53, 54]. First, the overall model fit compared to a randomized model was tested (ANOVA with 999 permutations). In case of a significant overall model, partial effects of the individual variables were tested (ANOVA by margin, 999 permutations). C:N ratio was excluded from multivariate analysis (Mantel test and CCA) as it was not measured independently.

### R packages

The R code is publicly available through https://github.com/VeraHesen. Package GUniFrac (v1.7) was used for calculation and visualisation of the rarefaction curves. Package metagenomeSeq (v1.14.0) was used to CSS normalize abundance count data. Package Car (v3.1-1) was used for type-II ANOVA. Package stats (v4.2.1) was used for Tukey HSD, MANOVA and calculation of Euclidian distance matrices. Package FactoMineR (v2.7) was used for PCA which was visualized with package factoextra (v1.0.7). Package MASS (v7.3-57) was used for LDA. Package ggVennDiagram (v1.2.2) was used for Venn diagrams. Package vegan (v2.6-4) was used for rarefaction of abundance count data, calculation of Bray-Curtis dissimilarity matrices, NMDS, PERMANOVA, Shannon *H* index, Mantel test and CCA. Package pairwiseAdonis (v0.4.1) was used for pairwise PERMANOVA. Figures were made with package ggplot2 (v3.4.0) and ggvegan (v0.1-0).

## Results

### *A. thaliana* plants collected from different succession classes grow in divergent abiotic soil compositions

**Fig. 1:**
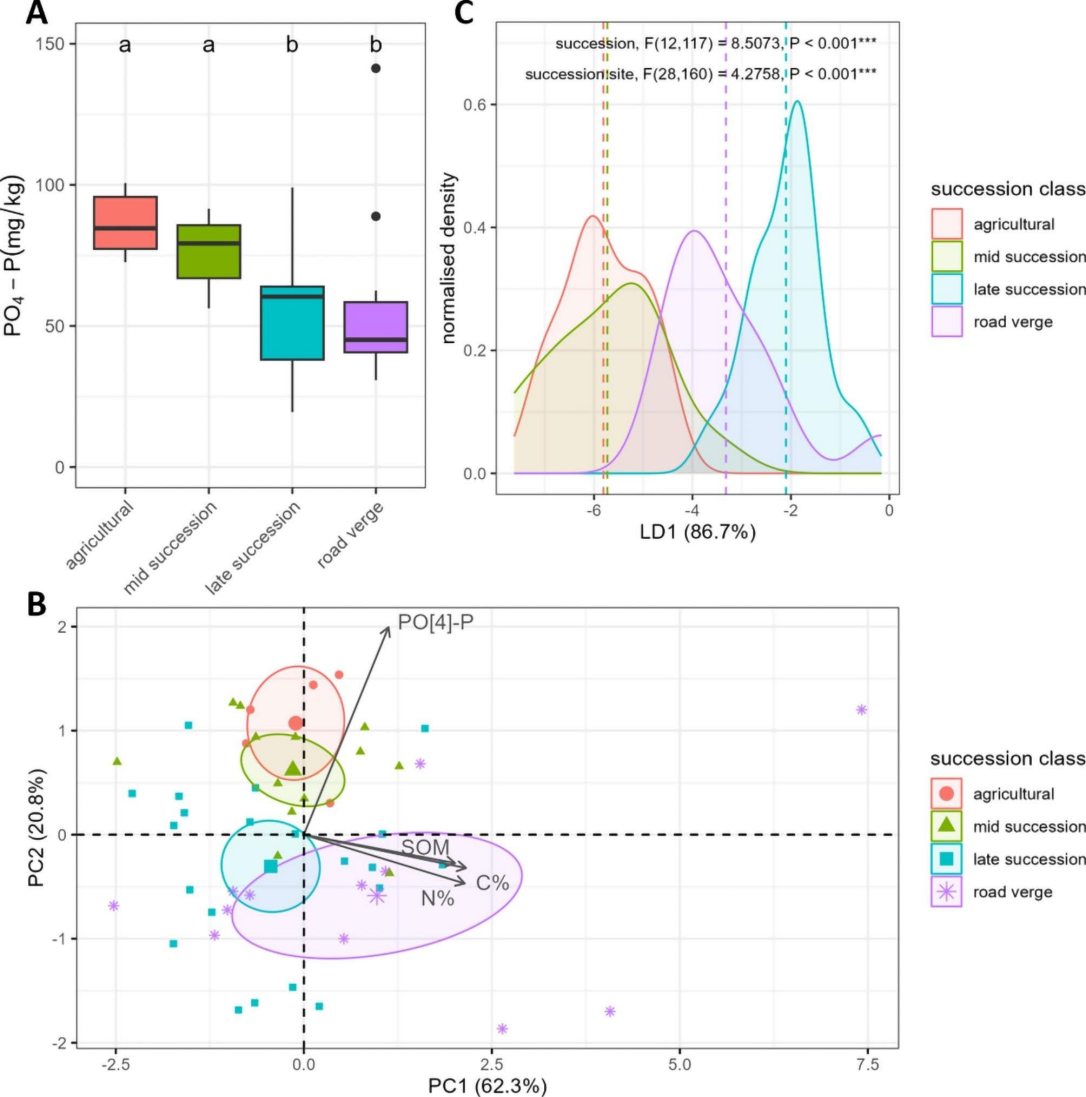
*Abiotic soil composition of the different succession classes. ***A**) *Boxplot of plant-available phosphate content (mg/kg) in soil from the different succession classes tested with type-II ANOVA. Letters indicate significant differences between succession classes based on Tukey HSD. ***B**) *PCA biplot of abiotic soil factors distinguishing the different succession classes. Small shapes indicate individual samples, and the large shapes indicate succession class means. Ellipsoids indicate 95% confidence intervals around the succession class mean. Arrows indicate relative contribution of independent soil factors to PC1 and PC2. ***C**) Linear discriminant analysis of abiotic soil factors displaying linear discriminant 1 on the x-axis and normalised density of the variance on y-axis. Dashed lines indicate succession class mean of LD1. MANOVA results are indicated in top of graph *P < 0.05; ** P < 0.01; *** P < 0.001.

We first addressed the local abiotic composition of the soil in which *A. thaliana* plants were growing. When studying the individual abiotic soil factors, most notably plant-available phosphate differed between the succession classes (*F* = 10.0387, *P* < 0.001). Plant-available phosphate was highest in agricultural and mid succession sites and lowest in late succession and road verge sites (Fig. 1A, Table S1 and S2). Succession also had a significant effect on the other abiotic soil factors, but differences between the succession classes were relatively smaller and classes less distinguishable (Fig. S3, Table S1 and S2).

Distinct clusters were observed when grouping the combined abiotic soil factors by succession class (Fig. 1B). The succession class means and corresponding 95% confidence ellipses differentiated agricultural and mid succession sites from late succession and road verge sites. The different succession classes were mostly separated in dimension 2, which explained 20.8% of all variation. Plant-available phosphate contributed most to dimension 2 when compared to all abiotic soil factors (90.9% of all variation in dimension 2). As already emerged from the clustering, succession indeed had a significant effect on the combined abiotic soil factors (*F*_12,117_ = 8.5073, *P* < 0.001). To distinguish succession classes from one another, we used succession as a classifier and maximized the variation between classes while minimizing the variation within classes. Linear discriminant 1 explained 86.7% of all variation and separated three groups: agricultural and mid succession class versus late succession class versus road verge class (Fig. 1C). Although the effect of succession class on the combined abiotic factors was evident, site also had a significant effect on the combined abiotic soil factors (*F*_28,160_ = 4.2758, *P* < 0.001). The high level of variation within both succession classes and sites was also visible when grouping the combined abiotic soil factors by site instead of succession class (Fig. S4). All in all, this shows that agricultural and mid succession sites were most similar in abiotic composition while late succession and road verge sites were distinct and that ultimately the observed succession effect was dependent on the sampled sites.

### Root-associated microbial community composition of *A. thaliana *differs between succession classes and this is most strongly displayed in fungal communities

Next, we investigated the bacterial and fungal community composition of *A. thaliana* root-associated microbiome. With sequencing, the mean number of raw reads obtained per sample was 67,943 ± 3244 for bacteria (max = 139,186; min = 13,287) and 25,345 ± 1651 for fungi (max = 60,436; min = 2000). In total, our data sets contained 18,104 bacterial ASVs and 4596 fungal OTUs. Rarefaction curves of all bacterial and fungal samples are shown in Fig. S5A and Fig. S5B, respectively.

First, we checked how many bacterial ASVs and fungal OTUs were shared and how many were unique for the succession classes. The bacterial community shows that the majority of ASVs are unique for the various succession class only having a core of 9% of the ASVs present in all succession classes (Fig. S6A). On the contrary, the fungal community shows more overlap between the succession classes (core of 18%) and little unique and overlapping OTUs in agricultural sites (Fig. S6B). Next, we evaluated phyla abundances and whether these are affected by succession. In total, our data sets contained 42 bacterial phyla and 12 fungal phyla. The most abundant bacterial phylum across all succession classes was *Proteobacteria* (30.95%), followed by *Actinobacteriota* (23.66%) and *Acidobacteriota* (9.09%) (Fig. S7A). The majority of the fungal OTUs across all succession classes belonged to the phylum of *Ascomycota* (65.39%), followed by *Basidiomycota* (14.79%) and *Mortierellomycota* (2.99%) (Fig. S7B). Neither highly abundant bacterial nor highly abundant fungal phyla showed major shifts between the succession classes (Fig. S7). To complement these findings, we studied the 10 most abundant bacterial and fungal taxa irrespective of their taxonomy. The 10 most abundant bacterial ASVs accounted for 8.33% of the total amount of reads and comprised of a relatively large number of phylum *Proteobacteria*, more specifically order *Rhizobiales* and family *Xanthobacteraceae* (Additional file 6). The 10 most abundant bacterial taxa vary in their presence in the different succession classes (Fig. S8A). The 10 most abundant fungal OTUs accounted for 22.03% of the total amount of reads and comprised mostly of phylum *Ascomycota* although the annotation is poor (Additional file 6). The majority of the 10 most abundant fungal OTUs seem to be less present in agricultural sites than in other succession classes while the third most abundant fungal OTU (species *Nadsonia_commutata_SH1570251.08FU)* is almost exclusively present in agricultural sites (Fig. S8B).

Besides the taxonomic evaluation, we assessed the differences in species richness between the succession classes. In bacteria, there was no significant effect of succession on species richness (*F* = 0.9842, *P* = 0.410; Table S3, Fig. S9A). On the contrary, succession did have a significant effect on fungal species richness (*F* = 7.0469, *P* = 0.0007582; Table S3, Fig. S9B). Fungal species richness was significantly lower in agricultural sites compared to mid succession sites, late succession sites and road verge sites (respectively *P* = 0.0101, *P* = 0.0111 and *P* = 0.0003; Table S4).

**Fig. 2:**
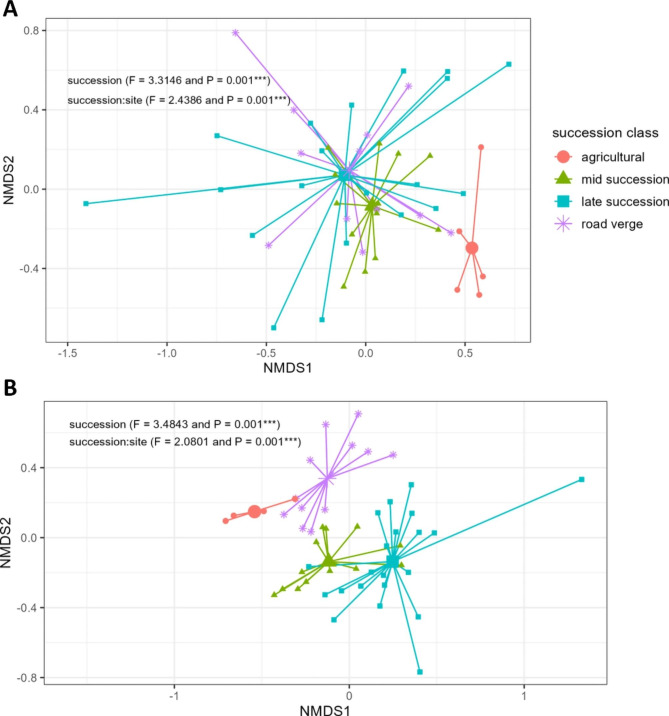
*Between-sample (beta) diversity of the root-associated bacterial and fungal communities showing succession class variation. ***A**) *NMDS on bacterial between-sample diversity*. *Dimensions = 3 and Stress = 0.125. ***B**) NMDS of fungal between-sample diversity. Dimensions = 3 and Stress = 0.161. Individual spiders per succession classes with small shapes indicating individual samples and the large shapes the succession class mean. PERMANOVA results are indicated in top of each plot *P < 0.05; ** P < 0.01; *** P < 0.001

When considering the between-sample diversity in composition, both bacteria and fungi showed distinct clusters according to succession classes (Fig. 2). Succession significantly explained the bacterial community composition differences (*F* = 3.3146 and *P* = 0.001; Table S5). The bacterial community composition of late succession and road verge sites could not be distinguished from one another as reflected in the non-significant pairwise comparison between these two succession classes (*F* = 1.3644, *P* = 0.112; Table S6). Nevertheless, all other pairwise comparisons between the bacterial community compositions of the different succession classes were significant (*P* < 0.01; Table S6). Succession significantly explained the fungal community composition and all pairwise comparisons between the succession classes were significant too (respectively *F* = 3.4843, *P* = 0.001 and *P* < 0.01; Table S5 and S6). In line with the statistical results, the fungal community composition showed a substantially clearer separation of succession class clusters than the bacterial community composition (Fig. 2).

We also analysed the variation within the succession classes and within sites for the bacterial and fungal community composition. Bacterial community composition showed a high level of variation within sites, thereby creating overlap between the succession classes (Fig. S10A). Fungal community composition showed less variation within sites and thereby maintained a clearer separation between the succession classes (Fig. S10B). Site had a significant effect on both bacterial (*F* = 2.4386, *P* = 0.001) and fungal (*F* = 2.0801, *P* = 0.001) community composition (Table S5).

Both the abiotic and microbial composition of *A. thaliana’s* root-associated soil showed similar differences according to succession class (Figs. 1 and 2). Therefore, we examined the interplay between the abiotic composition and bacterial or fungal community composition in more detail. There was a significant correlation between the bacterial community composition and the abiotic composition (Mantel R = 0.3367, *P* = 0.0088). However, the correlation was not significant between the fungal community composition and abiotic composition (Mantel R = 0.1166, *P* = 0.133). Besides the overall correlation between the abiotic and microbial composition, we investigated what part of the microbial community variation could be explained by the abiotic soil factors. Canonical Correspondence Analysis (CCA) revealed that 11.8% of the bacterial community composition could be explained by the abiotic soil factors (*F* = 1.4989, *P* = 0.001). Although there was no significant correlation in the overall fungal community composition and abiotic data, 10.6% of the community composition could be explained by abiotic soil factors (*F* = 1.3279, *P* = 0.002). In the constrained part of the bacterial community (11.8%), succession classes were mostly differentiated by soil organic matter content (Fig. S11A). In the constrained part of the fungal community (10.6%), succession classes were mostly differentiated by plant-available phosphate and soil organic matter content (Fig. S11B).

Succession class dependent differences are not obvious from phyla-level taxonomy nor species richness although fungal species richness is decreased in agricultural sites. When considering the overall community composition, both the bacterial and fungal community composition of *A. thaliana’s* root-associated differs depending on the succession class. Both succession and site had a significant effect on the community composition, showing that the effect of succession depends on the sampled sites. Interestingly, the overall differences between succession classes were more strongly displayed for fungi than for bacteria.

## Discussion

We examined the biotic and abiotic composition of the root-associated soil of *A. thaliana* along a succession gradient from agricultural fields to mid and late secondary succession sites and included road verges as an outgroup. As all sites were disturbed either by human management or animals, disturbance appeared to be an essential element for *A. thaliana* plants to appear in the vegetation. When this happened, *A. thaliana* was able to successfully establish populations in these variable soils. We were able to examine the different soil ecological contexts *A. thaliana* occurred in because the studied sites are within the same climatic, soil and (former) management conditions. For both soil bacteria and fungi, we could demonstrate that *A. thaliana* was able to grow in a wide variety of microbial communities on a local scale. However, we also realize that our study could have been constrained by the sample size and unbalanced design. Using a chronosequence as a space for time substitution limits the experimental design but also allowed us to conduct this research based on a well-established soil ecological basis. Interestingly, despite the limitations we were still able to detect clear succession dependent differences in both the abiotic and biotic composition of the *A. thaliana* microbiome. As this is a local study, it remains to be tested whether the finding that *A. thaliana* can span a succession gradient and thereby be exposed to a diverse microbiome is reflected in other secondary succession gradients.

The agricultural, mid and late succession sites form a secondary succession gradient while road verge sites may be considered an ecological outgroup. Interestingly, both microbial kingdoms, as well as the abiotic factors show a gradient where mid succession cluster is situated between the agricultural (its ecological preceding class) and late succession cluster (its ecological following class) (Figs. 1B and 2). Road verges resemble mostly the late succession class considering the bacterial communities and abiotic composition, while they form a separate group in the fungal community analysis (Figs. 1B and 2). How these microbial communities are different between the succession appears to differ between bacteria and fungi. For bacteria, we detected no differences in the species richness and saw an even distribution of unique and shared taxa between the succession classes (Fig. S9A and Fig. S6A). On the contrary, for fungi the compositional differences in agricultural sites compared to other succession classes might be partially explained by a reduced species richness and reduce number of unique and overlapping taxa in agricultural sites (Fig. S9B and Fig. S6B). For neither bacteria nor fungi there was a clear distinction of succession classes based on major phyla, suggesting that phyla level taxonomy is too much of a generalization when describing soil succession at this scale. The process of secondary succession is highly complex and a non-linear process by which both the vegetation and soil biota composition shifts, which influences one another [26, 27, 34, 55]. Succession is an ongoing process reflected in the years since disturbance. Yet, with the employed succession classification in our study we can show that *A. thaliana* spans this gradient and is thereby exposed to a variety of microbiome compositions from early to later secondary succession.

In addition to showing that *A. thaliana* can be exposed to a wide variety of bacterial and fungal community compositions, our results showed succession dependent discrepancies between the bacterial and fungal communities. This leads to the notion that the *A. thaliana* microbiome is poorly represented when only studying bacteria. Although the importance of fungi in processes such as nutrient cycling, symbiotic interactions and creation of microhabitats has been well acknowledged [56] investigating this kingdom suffered from a less complete taxonomic and functional annotation than is known for bacteria [57, 58]. Moreover, fungi have received little attention in *A. thaliana* microbiome research which has been focused on bacteria in the past decade [10, 12, 15] The benefit of studying fungi in addition to bacteria is exemplified in a recent study where they showed the relation between the fungal microbiome and *A. thaliana’s* genetics [59] and the body of work investigating the role of fungi in the *A. thaliana* microbiome continues to grow [60–62]. Including fungi and potentially even more soil microbes such as protists [17] will give a more complete understanding of plant-microbiome interactions.

Previous studies looking into the composition and selection strategies of the *A. thaliana* root-associated and core microbiome used natural soil inocula or sampled natural plant populations in their local environment [15–17, 28]. Most often these sites were highly disturbed, poor soils with considerable human-influence, for example road verges and agricultural fields. This type of *A. thaliana* environment is also covered in our research, but in addition, we showed that *A. thaliana* is able to naturally occur in a much wider soil ecological context than previously assumed for an early succession pioneer species. This finding is of eminent importance as it was demonstrated that *A. thaliana’s* root-associated microbiome mostly depends on the local bulk soil [10, 13]. However, we currently do not know whether or how the ability of *A. thaliana* to span a succession gradient could affect plant performance or even local adaptation. Therefore, it remains to be investigated what the implications of the wider soil ecological context and thereby diversified local bulk soil are for *A. thaliana*.

## Conclusions

In conclusion, studying the natural soil microbiome of *A. thaliana* within its ecological context reveals new opportunities for future plant-microbiome studies. Our study demonstrated the wide secondary succession context *A. thaliana* naturally occurs in and this can be combined with the wealth of mechanistic studies, molecular tools, and genetic resources of this molecular model plant species. Future plant-microbiome studies can utilize *A. thaliana* in more complex soil compositions than has been done so far and thereby improve our understanding of causal plant-microbiome interactions. These insights in plant responses to soil microbiome composition may be used to make agricultural crops perform better in less disturbed, and therefore healthier soils.

## Electronic supplementary material

Below is the link to the electronic supplementary material.

**Supplementary Material 1. Additional file 1** - sampling sites.xlsx: Succession class refers to classification of the site and YSA indicates the years since agricultural abandonment. Mid succession is considered 10 to 20 YSA and late succession is considered over 20 YSA. Sample size refers to the number of *A. thaliana* plants sampled per site. Sampling method refers to the method used to sample the *A. thaliana* plants

**Supplementary Material 2. Additional file 2** - sampling info.docx: Document gives detailed information about the sampled sites and the methods used to sample the sites. Document provides a map of South Veluwe area with the sampling sites (Fig. S1) and illustrative pictures of the sampling (Fig. S2)

**Supplementary Material 3. Additional file 3** - abiotic data.csv: Raw abiotic soil factor data used for the abiotic soil analysis

**Supplementary Material 4. Additional file 4** - sequencing info.xlsx. Sample name, unique sample code (used in raw sequencing data) and their specific barcode sequence in reverse primer for the bacterial 16 S rRNA gene and fungal ITS2 region amplification are indicated

**Supplementary Material 5. Additional file 5** – Supplresults.docx: Documents provides all supplementary Tables (S1–6) and all supplementary Figs. (S3–11)

**Supplementary Material 6. Additional file 6** – 10 MA bacteria and fungi.xlsx. Overview of the 10 most abundant bacterial ASVs (sheet 1) and fungal OTUs (sheet 2) indicating relative abundance as percentage of total normalized reads and taxonomy. Ranking in abundance goes from top (number 1) to bottom (number 10). % is percentage of normalized reads of total amount of normalized reads in the dataset. The 10 most abundant bacterial OTUs account for 8.33% of total amount normalized reads in the whole data set. The 10 most abundant fungal OTUs account for 22.03% of total amount normalized reads in the whole data set

## Data Availability

Abiotic soil factor data is provided in Additional file 3 as csv file. The raw sequencing data of 16 S rRNA gene sequences and ITS2 sequences has been submitted to the European Nucleotide Archive (ENA) under accession PRJEB59993. The R code is publicly available through https://github.com/VeraHesen.
