# Supplementary material for: Pioneer Arabidopsis thaliana spans the succession gradient revealing a diverse root-associated microbiome
Source: Environ Microbiome. 2023 Jul 19;18:62. doi: 10.1186/s40793-023-00511-y (PMC10357733; doi:10.1186/s40793-023-00511-y)
Supplement: Supplementary file 2 — Supplementary Material 2. Additional file 2 - sampling info.docx: Document gives detailed information about the sampled sites and the methods used to sample the sites. Document provides a map of South Veluwe area with the sampling sites (Fig. S1) and illustrative pictures of the sampling (Fig. S2) [file 40793_2023_511_MOESM2_ESM.docx]

**Pioneer *Arabidopsis thaliana* spans the succession gradient revealing a diverse root-associated microbiome**

Vera Hesen^1,2^, Yvet Boele^1^, Tanja Bakx-Schotman^2^, Femke van Beersum^2,3^, Ciska Raaijmakers^2^, Ben Scheres^1,4^, Viola Willemsen^1^, Wim H. van der Putten^2,5^

1 Cluster of Plant Developmental Biology, Laboratory of Molecular Biology, Wageningen University, Droevendaalsesteeg 1, 6708 PB Wageningen, the Netherlands

2 Department of Terrestrial Ecology, Netherlands Institute of Ecology (NIOO-KNAW), Droevendaalsesteeg 10, 6700 AB Wageningen, the Netherlands

3 Plant Ecology and Nature Conservation Group, Wageningen University, Droevendaalsesteeg 3a, 6708 PB Wageningen, the Netherlands

4 Rijk Zwaan Breeding B.V., Department of Biotechnology, Eerste Kruisweg 9, 4793 RS Fijnaart, the Netherlands

5 Laboratory of Nematology, Wageningen University, Droevendaalsesteeg 1, 6708 PB Wageningen, the Netherlands

**Detailed description of sites and sampling methodology**

The 11 sampling sites are all located in the South Veluwe region (Fig. S1). This region is composed of soil originating from glacial sand deposits. The agricultural and former agricultural sites have (had) similar agricultural practices. The former agricultural sites are located in protected areas and were sampled with permission of the local authorities. The agricultural sites undergo standard agricultural management such as ploughing, harvesting and application of external inputs. The former agricultural mid and late succession fields are dominated by grasses and experience animal activity (e.g., wild boars, hares, and ants). The road verge sites receive occasional mowing but no additional human management. Figure S2 shows illustrative pictures of the different succession classes as well as local *Arabidopsis thaliana* environment where the plants were sampled.
 Natural populations of *A. thaliana* were present on all sites. Plants were always found on disturbed sandy patches of soil. Depending on the succession class, this could be caused by human management or animal activity. In most sites, *A. thaliana* was overly abundant. In case of highly abundant *A. thaliana* presence, plants were sampled according to a transect. For the (former) agricultural sites, the start of the transect was 10 meters from the border of the field and the transect ran in a straight line and perpendicular fashion to the border of the field. For the road verge sites, the transect ran parallel to the road, on 1 meter distance. Depending on the density of the *A. thaliana* plants the length of the transect varied and was at least 10 meter to ensure a proper distance between the individual sample. Along the transect, 6 plants were collected on even distances. At the start of the transect a GPS measurement was made and distances between all plants were measured and noted. In case the *A. thaliana* plants were less abundant, 6 suitable plants were found at the site in an area of at least 40 m^2^ to ensure a proper distance between the individual samples. At the position of each individual plant GPS measurements were taken. At all 11 sampling sites 6 plants and corresponding root-associated soil sample and bulk soil sample were collected, but due to technical reasons the number of individual samples reduced to a final 51 samples.


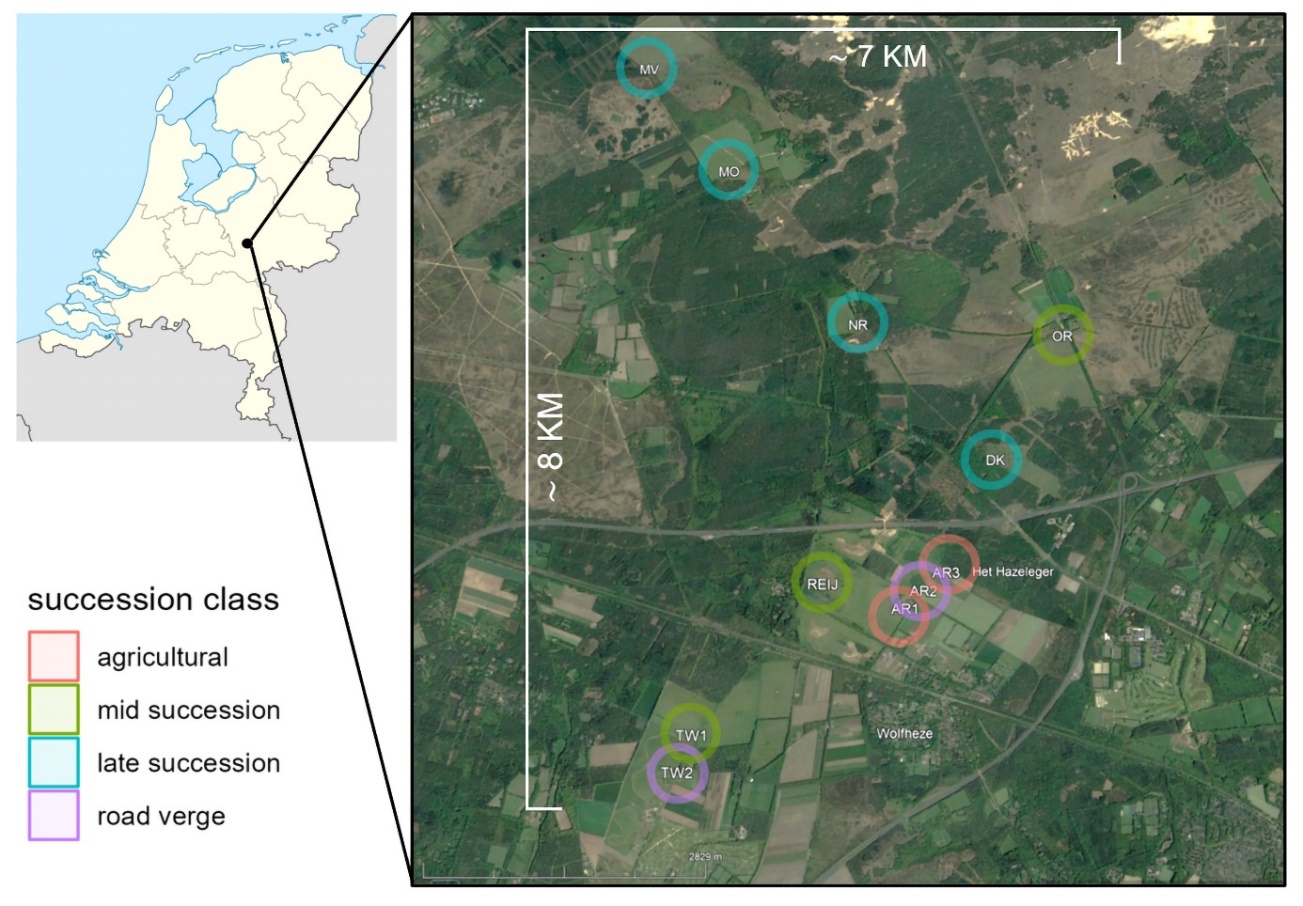

**Figure S1 |** *Map of the South Veluwe area indicating the 11 sampling sites. Colours indicate succession classes. Inset depicts location of South Veluwe are in the Netherlands.*


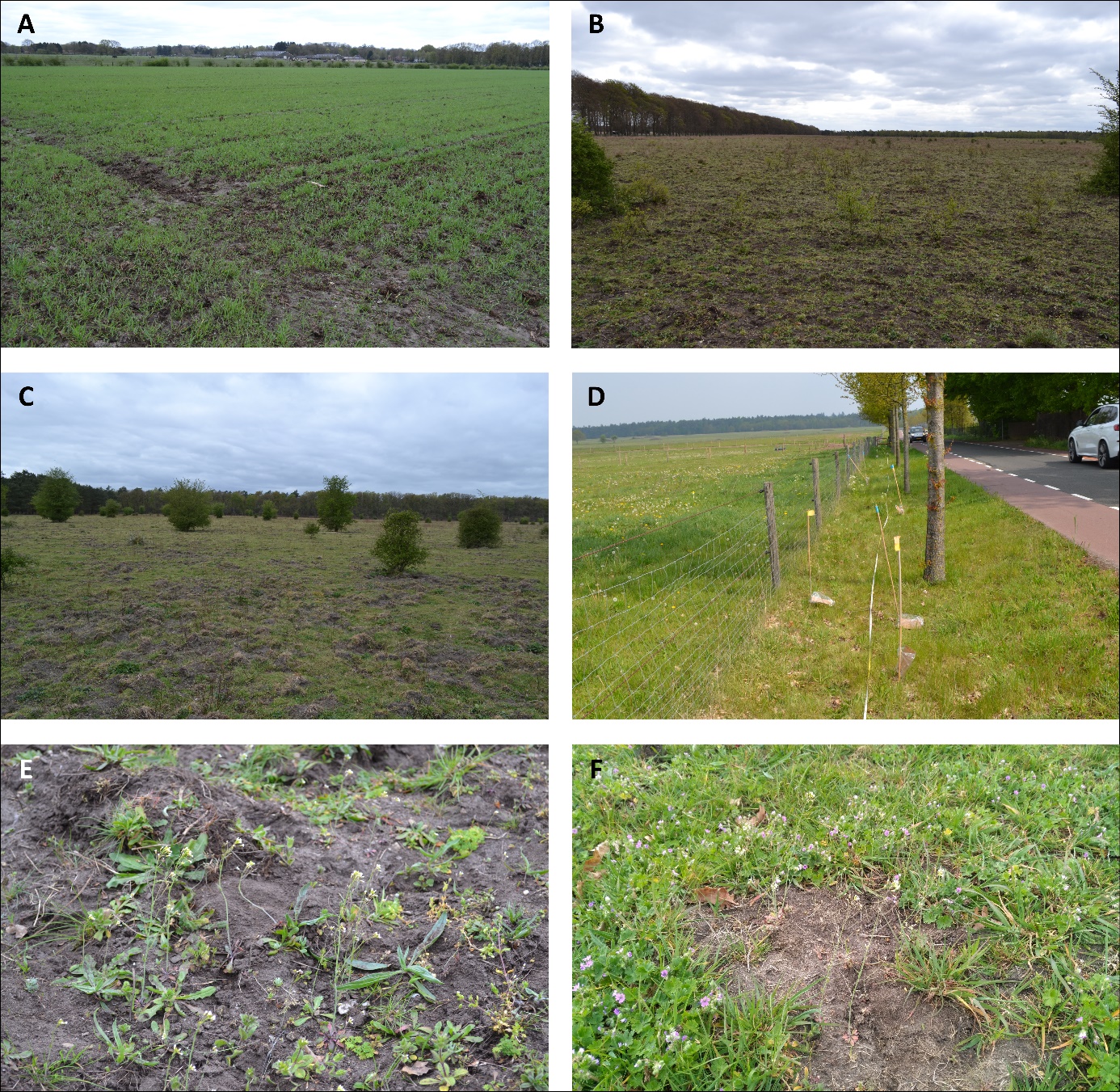


**Figure S2 |** *Pictures of the different succession class sites and local environment of the sampled A. thaliana plants.* **A)** *Picture of Akker Reijerskamp 1, agricultural succession class.* ***B)*** *Picture of Oud Reemst, mid succession class.* **C)** P*icture of Nieuw Reemst, late succession class.* **D)** *Picture of Telefoonweg 2, road verge succession class*. **E)** *Example of local A. thaliana environment at Oud Reemst.* ***F)*** *Example of local A. thaliana environment at Telefoonweg 1.*
