# Supplementary material for: Pioneer Arabidopsis thaliana spans the succession gradient revealing a diverse root-associated microbiome
Source: Environ Microbiome. 2023 Jul 19;18:62. doi: 10.1186/s40793-023-00511-y (PMC10357733; doi:10.1186/s40793-023-00511-y)
Supplement: Supplementary file 5 — Supplementary Material 5. Additional file 5 – Supplresults.docx: Documents provides all supplementary Tables (S1–6) and all supplementary Figs. (S3–11) [file 40793_2023_511_MOESM5_ESM.docx]

**Pioneer *Arabidopsis thaliana* spans the succession gradient revealing a diverse root-associated microbiome**

Vera Hesen^1,2^, Yvet Boele^1^, Tanja Bakx-Schotman^2^, Femke van Beersum^2,3^, Ciska Raaijmakers^2^, Ben Scheres^1,4^, Viola Willemsen^1^, Wim H. van der Putten^2,5^

1 Cluster of Plant Developmental Biology, Laboratory of Molecular Biology, Wageningen University, Droevendaalsesteeg 1, 6708 PB Wageningen, the Netherlands

2 Department of Terrestrial Ecology, Netherlands Institute of Ecology (NIOO-KNAW), Droevendaalsesteeg 10, 6700 AB Wageningen, the Netherlands

3 Plant Ecology and Nature Conservation Group, Wageningen University, Droevendaalsesteeg 3a, 6708 PB Wageningen, the Netherlands

4 Rijk Zwaan Breeding B.V., Department of Biotechnology, Eerste Kruisweg 9, 4793 RS Fijnaart, the Netherlands

5 Laboratory of Nematology, Wageningen University, Droevendaalsesteeg 1, 6708 PB Wageningen, the Netherlands

**Supplementary results**

| Succession class | SOM  (g/100g dry soil) | | PO_4_-P  (mg/kg dry soil) | | C  (%) | | N  (%) | | C:N | |
| --- | --- | --- | --- | --- | --- | --- | --- | --- | --- | --- |
| Agricultural | 6.25 | ±0.55 | 86.17 | ±5.32 | 3.92 | ±0.33 | 0.20 | ±0.02 | 19.09 | ±0.50 |
| Mid succession | 5.88 | ±0.24 | 76.04 | ±3.17 | 4.25 | ±0.23 | 0.22 | ±0.01 | 19.47 | ±0.33 |
| Late succession | 5.88 | ±0.21 | 54.00 | ±4.43 | 3.80 | ±0.20 | 0.24 | ±0.01 | 15.42 | ±0.26 |
| Road verge | 7.12 | ±0.55 | 56.95 | ±8.87 | 4.88 | ±0.46 | 0.29 | ±0.03 | 17.40 | ±0.60 |

**Table S1 |** *Overview of the soil abiotic composition of the four succession classes. Reported values are means with corresponding standard error.*

**Table S2 |** *Effect of succession class and site nested within succession class on abiotic soil factors. This is tested using type-II ANOVA (abiotic soil factor ~ succession / site). Values in bold represent significant effects with P < 0.05, Df indicates Degrees of freedom, * P < 0.05; ** P < 0.01; *** P < 0.001.*

| Response variable |  | Df | *F*-value | *P*-value |
| --- | --- | --- | --- | --- |
| SOM | succession | 3 | 6.3602 | **0.001254 **** |
|  | succession:site | 7 | 8.1967 | **3.594e-06 ***** |
| PO_4_-P | succession | 3 | 10.0387 | **4.629e-05***** |
|  | succession:site | 7 | 6.9844 | **1.902e-05***** |
| C% | succession | 3 | 3.7405 | **0.018474 *** |
|  | succession:site | 7 | 3.5064 | **0.005021 **** |
| N% | succession | 3 | 3.8494 | **0.0164171 *** |
|  | succession:site | 7 | 4.7808 | **0.0005619 ***** |
| C:N | succession | 3 | 71.617 | **3.902e-16 ***** |
|  | succession:site | 7 | 14.320 | **3.813e-09 ***** |

**Table S3 |** *Effect of succession class and site nested within succession class on bacterial and fungal species richness. This is tested using type-II ANOVA (Shannon H index ~ succession / site). Values in bold represent significant effects with P < 0.05, Df indicates Degrees of freedom, * P < 0.05; ** P < 0.01; *** P < 0.001*.

| Response variable |  | Df | *F*-value | *P*-value |
| --- | --- | --- | --- | --- |
| Bacterial Shannon *H* index | succession | 3 | 0.9842 | 0.410739 |
|  | succession:site | 7 | 4.4302 | **0.001167**** |
| Fungal Shannon *H* index | succession | 3 | 7.0469 | **0.0007582***** |
|  | succession:site | 7 | 2.3378 | **0.0417292*** |

**Table S4 |** *Pairwise comparisons between succession classes on fungal species richness. This is tested using TukeyHSD. Values in bold represent significant effects with P < 0.05, * P < 0.05; ** P < 0.01; *** P < 0.001.*

| Fungal Shannon  *H* index | Pairs | *P*-value adj. |
| --- | --- | --- |
|  | road verge vs. mid succession | 0.2270749 |
|  | road verge vs. late succession | 0.1048878 |
|  | road verge vs. agricultural | **0.0003590***** |
|  | mid succession vs. late succession | 0.9947078 |
|  | mid succession vs. agricultural | **0.0100914*** |
|  | late succession vs. agricultural | **0.0111687*** |

**Table S5 |** *Effect of succession class and site nested within succession class on between-sample diversity of bacterial ASVs and fungal OTUs. This is tested by using PERMANOVA (Bray-Curtis dissimilarity matrix ~ succession / site) with 999 permutations. Values in bold represent significant effects with P < 0.05, Df indicates Degrees of freedom, * P < 0.05; ** P < 0.01; *** P < 0.001.*

| Response variable |  | Df | *F*-value | *P*-value |
| --- | --- | --- | --- | --- |
| Bacterial ASVs | succession | 3 | 3.3146 | **0.001 ***** |
|  | succession:site | 7 | 2.4386 | **0.001 ***** |
| Fungal OTUs | succession | 3 | 3.4843 | **0.001 ***** |
|  | succession:site | 7 | 2.0801 | **0.001 ***** |

**Table S6 |** *Pairwise comparisons between succession classes on between-sample (beta) diversity of bacterial ASVs and fungal OTUs. This is tested using PERMANOVA (Bray-Curtis dissimilarity matrix ~ succession / site) with 999 permutations. P-value adjusted using Benjamini-Hochberg for multiple testing. Values in bold represent significant effects with P < 0.05, Df indicates Degrees of freedom, * P < 0.05; ** P < 0.01; *** P < 0.001.*

| Response variable | Pairs | Df | *F*-value | *P*-value adj. |
| --- | --- | --- | --- | --- |
| Bacterial ASVs | road verge vs. mid succession | 1 | 2.33643 | **0.0015**** |
|  | road verge vs. late succession | 1 | 1.364448 | 0.112 |
|  | road verge vs. agricultural | 1 | 3.920617 | **0.0015**** |
|  | mid succession vs. late succession | 1 | 2.463162 | **0.0024**** |
|  | mid succession vs. agricultural | 1 | 3.81912 | **0.0015**** |
|  | late succession vs. agricultural | 1 | 3.901893 | **0.0015**** |
| Fungal OTUs | road verge vs. mid succession | 1 | 2.761676 | **0.002**** |
|  | road verge vs. late succession | 1 | 2.879826 | **0.002**** |
|  | road verge vs. agricultural | 1 | 4.004174 | **0.003**** |
|  | mid succession vs. late succession | 1 | 1.907604 | **0.003**** |
|  | mid succession vs. agricultural | 1 | 3.544225 | **0.003**** |
|  | late succession vs. agricultural | 1 | 3.914776 | **0.002**** |

**
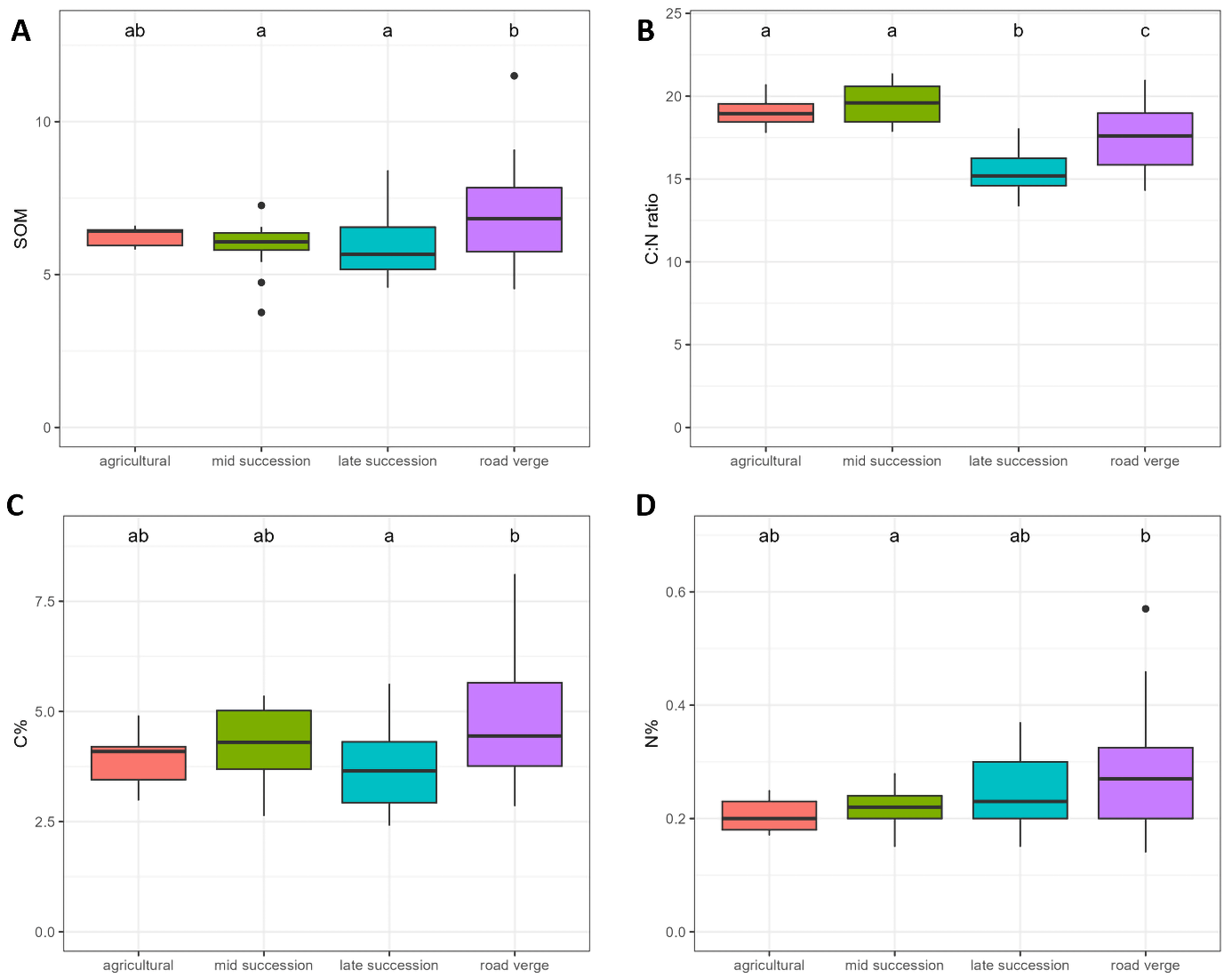
**

**Figures S3 |** *Abiotic soil factors for different succession classes.* **A**) *Soil organic matter content (g/100g dry soil).* **B**) *Nitrogen content (%).* **C**) *Carbon content (%).* **D**) *Carbon to Nitrogen ratio. Tested using type-II ANOVA. The same letter (either a or b) above the succession classes indicates that these groups are not significantly different based on Tukey HSD. If two succession classes do not share the same letter, these groups are significantly different based on Tukey HSD.*

*
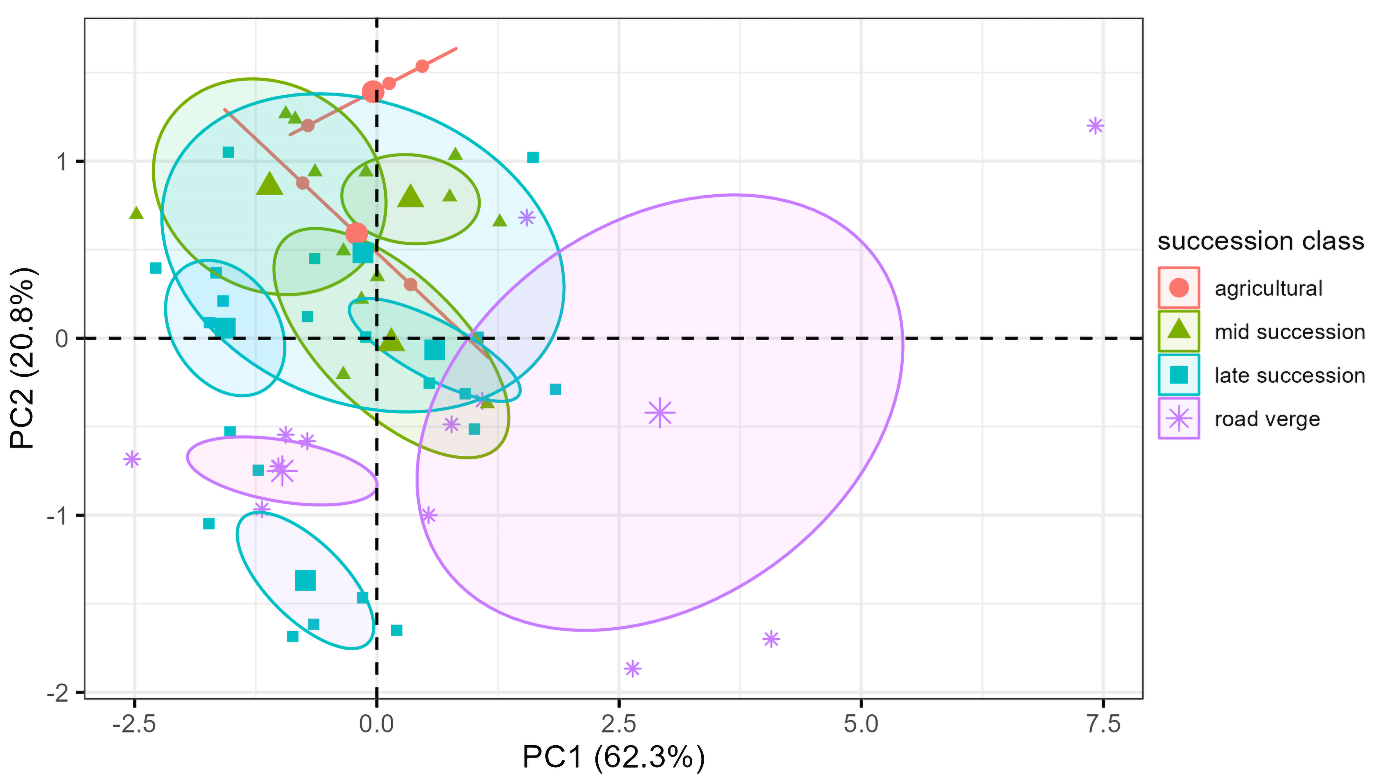
*

**Figure S4 |** *PCA of abiotic soil factors with clusters per individual site. Colours and shape indicate succession classes. Small shapes indicate individual samples, and the larger shapes indicate site means. Ellipsoids indicate 95% confidence intervals around the site mean.*

*
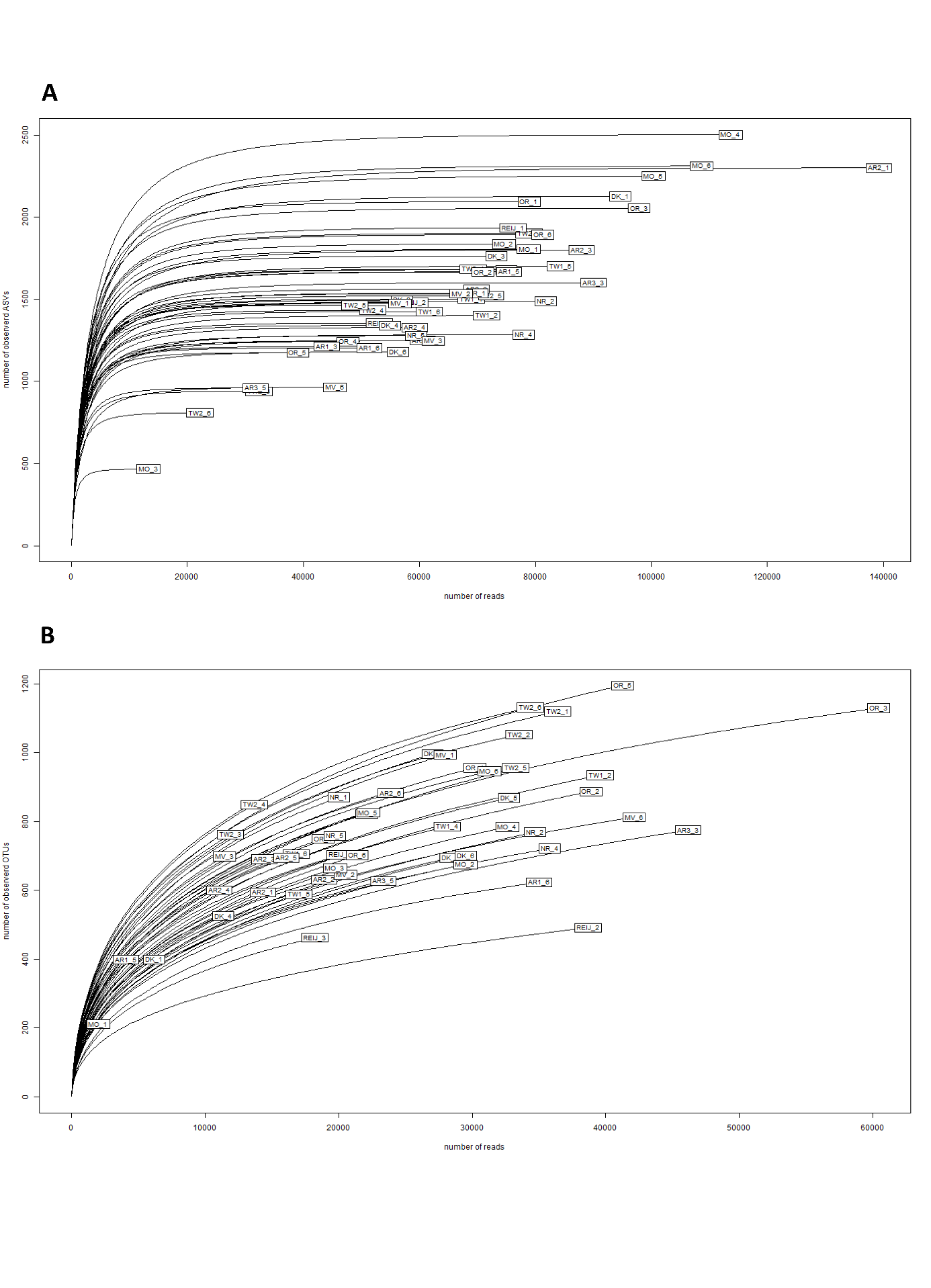
*

**Figure S5 |** *Rarefaction curves showing how well sequencing captured diversity per sample.* **A)** *16S bacterial samples; sequencing depth versus number of ASVs* **B)** *ITS fungal samples; sequencing depth versus number of OTUs.*

*
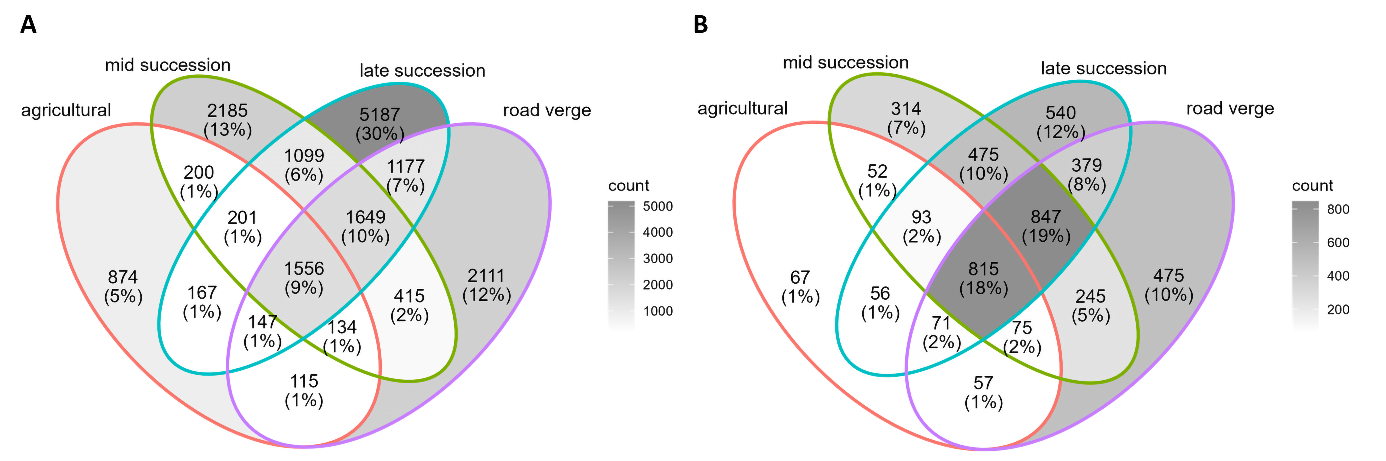
*

**Figure S6 |** *Number of unique and overlapping taxa between the succession classes.* **A)** *Bacterial ASVs.* **B)** *Fungal OTUs. Numbers in the Venn diagram indicate the ASVs or OTUs in count and in percentage. Shading represents the count.*

*
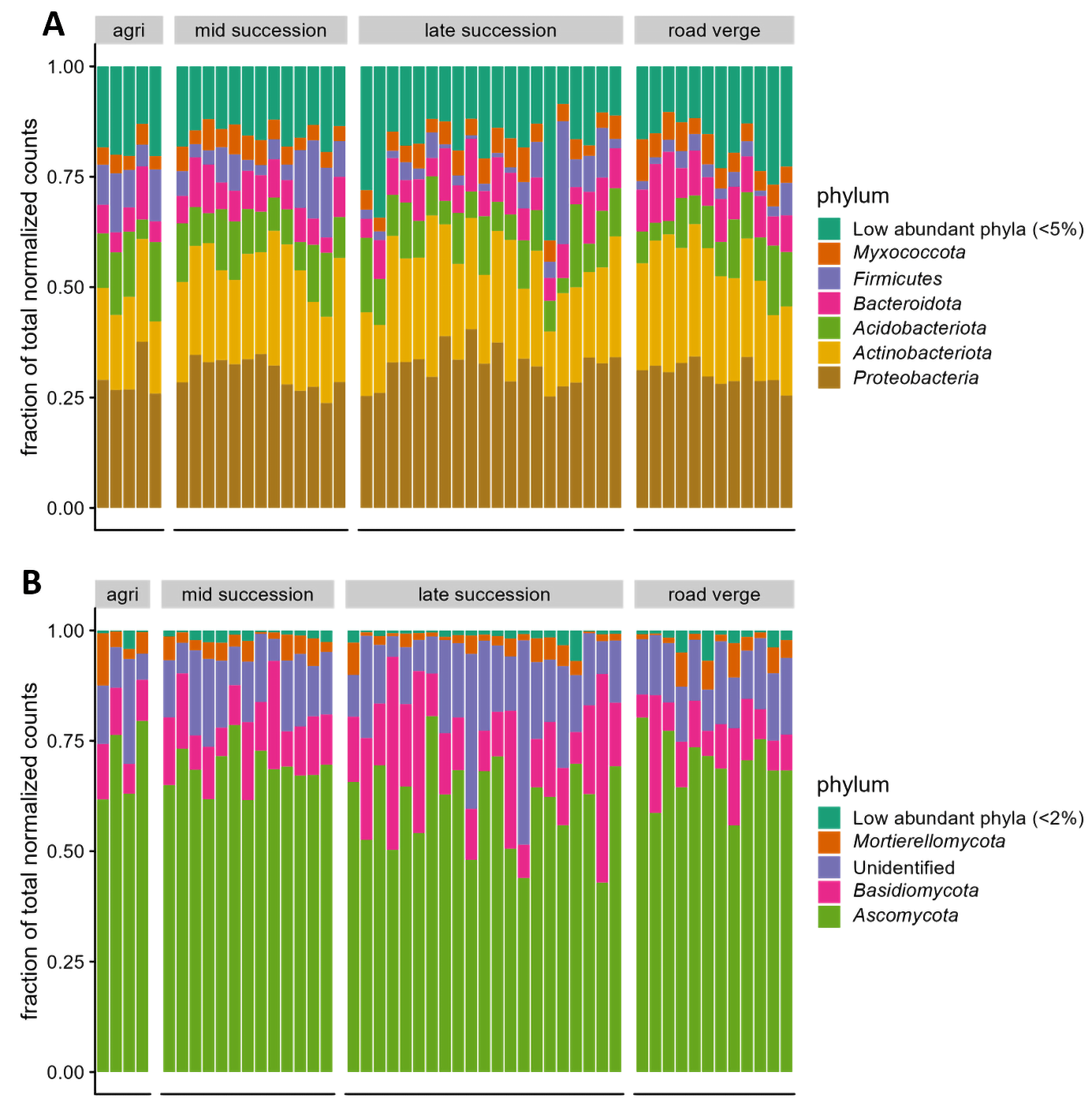
*

**Figure S7 |** *Most highly abundant phyla per succession class.* **A**) *Bacterial phyla, top 6 phyla displayed as only phyla with higher relative abundance than 5% are shown.* **B**) *Fungal phyla, top 3 phyla displayed as only phyla with higher relative abundance than 2% are shown. Abundance percentages are calculated per succession class. Individual bars are individual samples. Agri is abbreviation for agricultural succession class.*

*
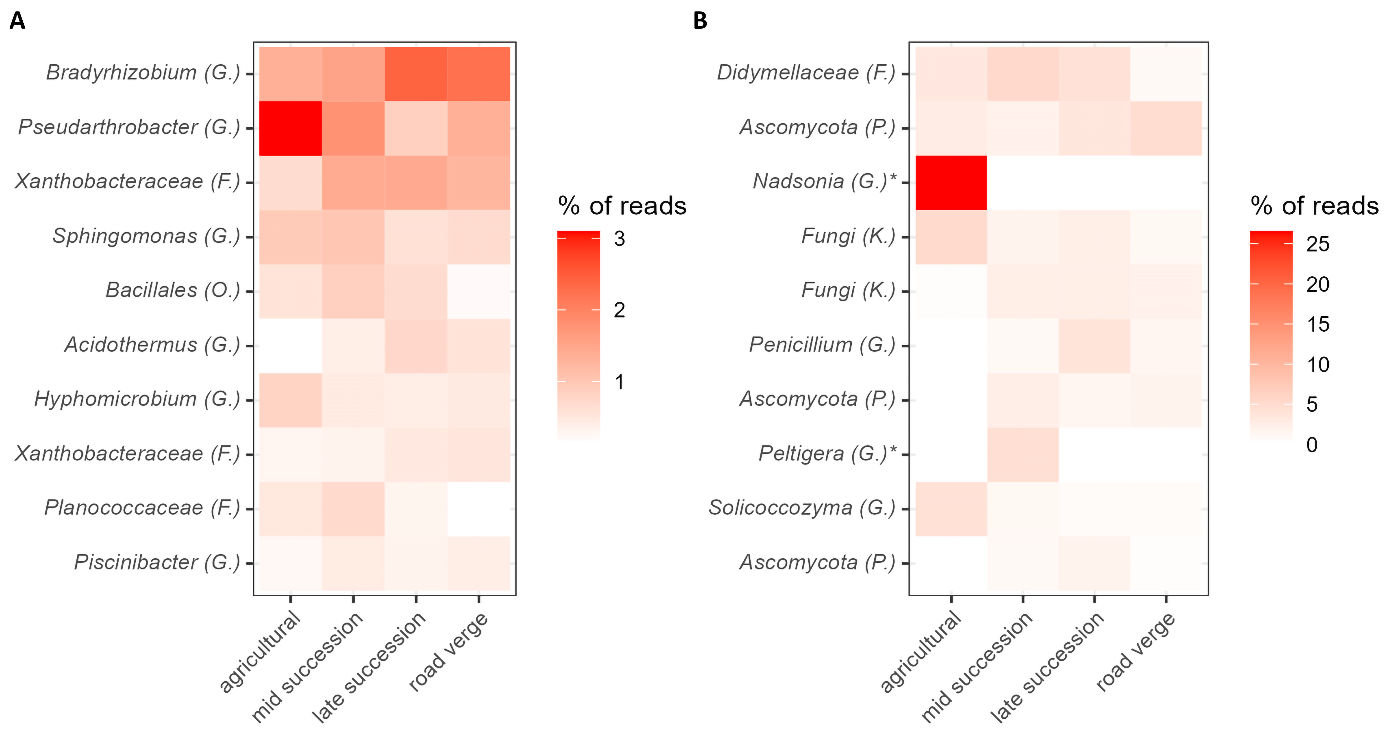
*

**Figure S8 |** *Heatmap depicting the percentage of reads of the 10 most abundant OTUs per succession class.* **A**) *10 most abundant bacterial ASVs.* *In total they account for 8.33% of the total amount of reads in the bacterial dataset* **B**) *10 most abundant fungal OTUs. In total they account for 22.03% of the total amount of reads in the fungal dataset. Ranking in abundance goes from top (number 1) to bottom (number 10). Percentages were calculated per succession class by dividing ASV or OTU read total per succession class by total number of reads in the respective succession class. K. indicates kingdom, P. indicates phylum, O. indicates order, F. indicates family and G. indicates genus. *For these taxa species level is known but due to readability of the plot not listed here.*

**
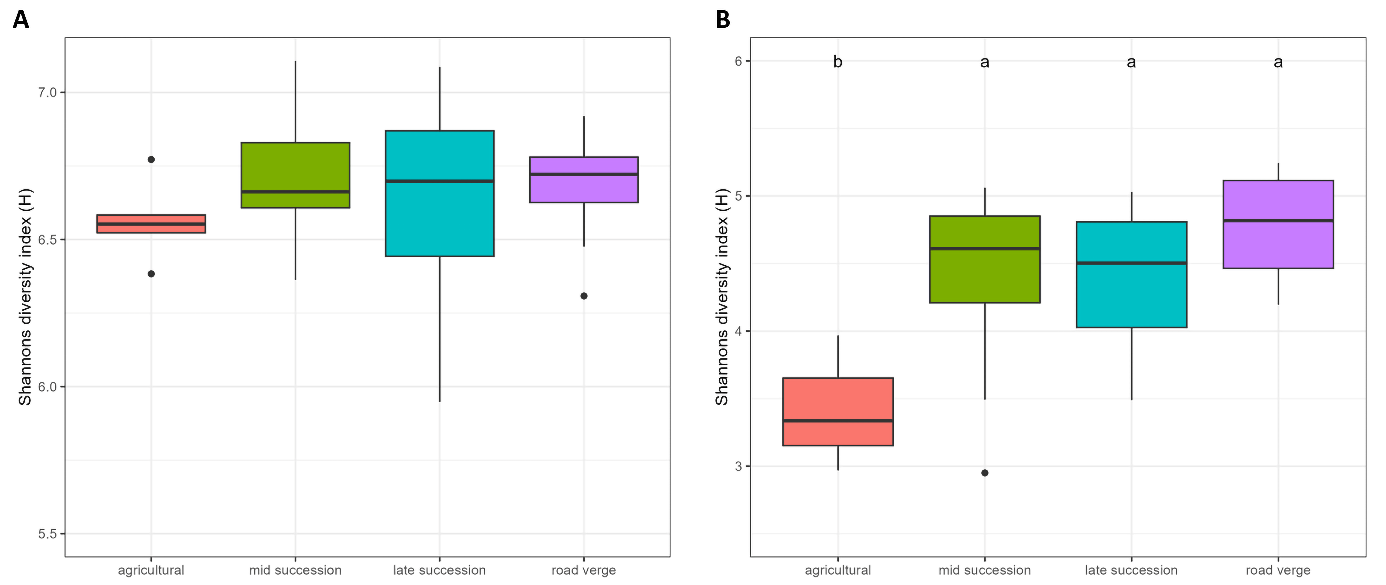
Figure S9 |** *Species richness for different succession classes represented by Shannon H index* **A**) *Bacterial species richness.* **B**) *Fungal species richness. Tested using type-II ANOVA. The same letter (either a or b) above the succession classes indicates that these groups are not significantly different based on Tukey HSD. If two succession classes do not share the same letter, these groups are significantly different based on Tukey HSD.*


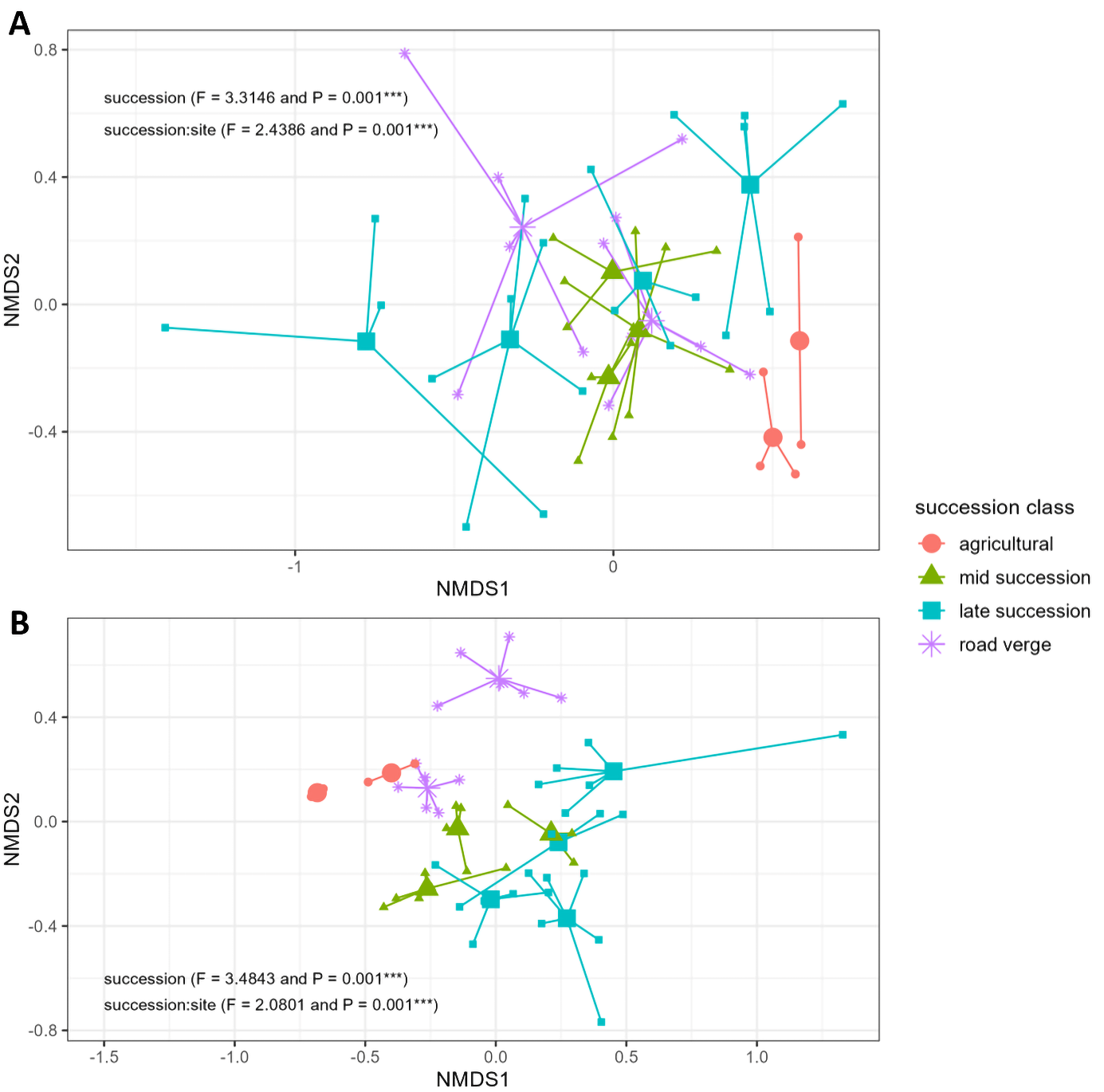

**Figure S10 |** *Between-sample (beta) diversity of the root-associated bacterial and fungal community showing site variation* **A**) *NMDS on bacterial between-sample diversity.* **B**) *NMDS of fungal between-sample diversity. Individual spiders per site with small shapes indicating individual samples and the large shapes the site mean. PERMANOVA results indicated in top and bottom of graph *P < 0.05; ** P < 0.01; *** P < 0.001.*


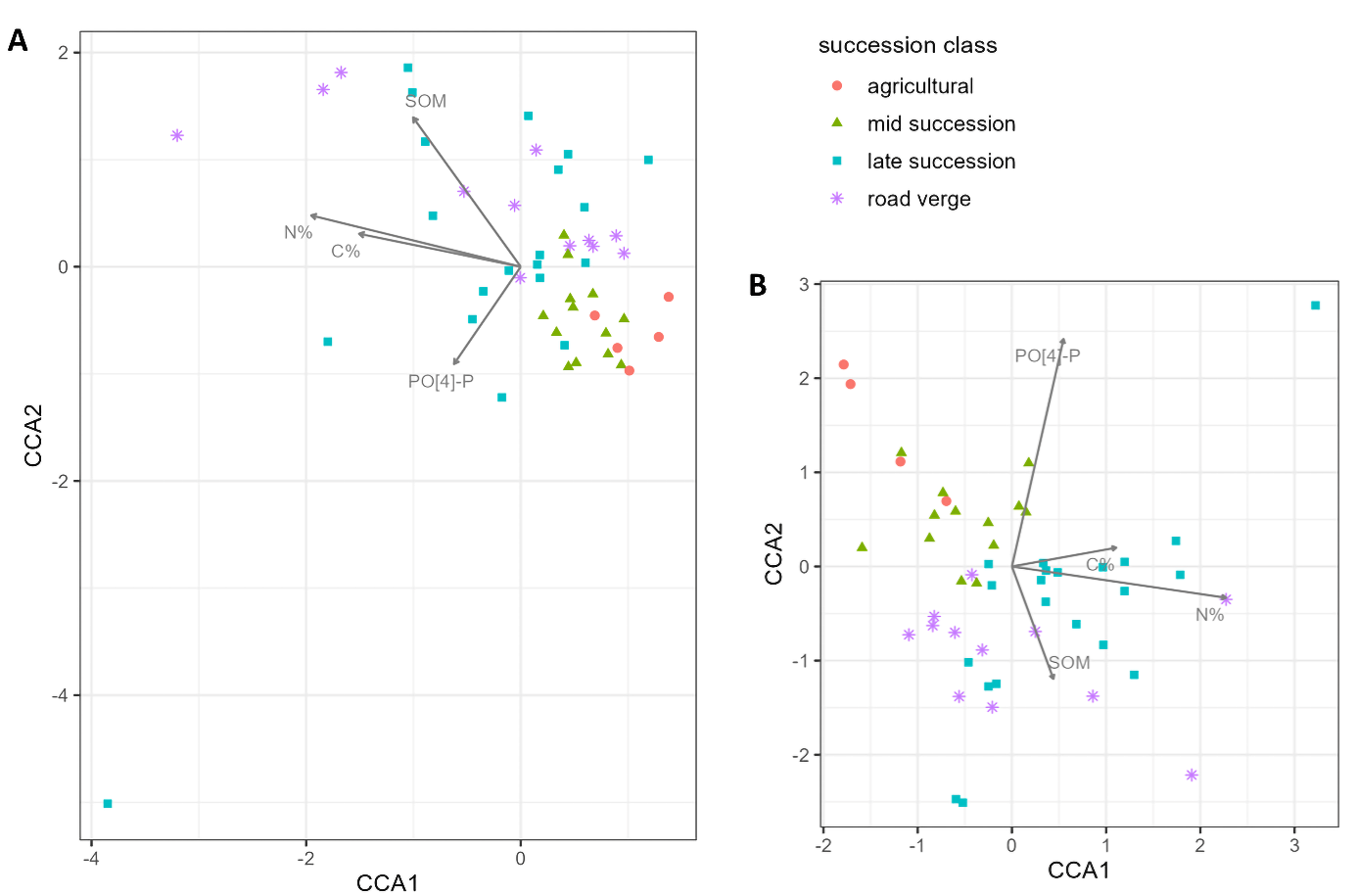

**Figure S11 |** *Constrained part of the community composition variation which can be explained by the abiotic soil factors.* **A**) *11.8% of the bacterial community composition variation.* **B**) *10.6% of the fungal community composition variation. Biplot showing individual samples and abiotic soil factor correlations (arrows).*
